# Adenomatoid odontogenic tumor associated with dentigerous cyst in posterior maxilla: A case report and review of literature

**DOI:** 10.4103/0973-029X.72502

**Published:** 2010

**Authors:** J Baby John, Reena Rachel John

**Affiliations:** *Johns Dental Speciality Centre, West Main Road, Mettur Dam, Salem, Chennai, India*

**Keywords:** Adenomatoid odontogenic tumor, dentigerous cyst, impacted tooth

## Abstract

Adenomatoid odontogenic tumor (AOT)-a benign (hamartomatous) lesion of odontogenic origin-is an uncommon tumor which affects young individuals with a female predominance, mainly in the second decade. This lesion is most commonly located in the anterior maxilla and is usually associated with an impacted canine tooth. This is a case report of a 39-year-old female patient presented with a large AOT of the posterior maxilla associated with an impacted second molar – a very rare situation.

## INTRODUCTION

Adenomatoid odontogenic tumor (AOT), an uncommon benign epithelial lesion of odontogenic origin was first described by Dreibaldt in 1907 as a pseudoadenoameloblastoma.[1,2] A number of terms were used to describe the tumor. Unal *et al*.[3] produced a list containing all nomenclatures for AOT reported in the literatures. Terms such as adeno ameloblastoma, ameloblastic adenomatoid tumor, adamantinoma, epithelioma adamantinum or teratomatous odontoma have been used before to define the lesion which is currently known as AOT. It was Stafne in 1948 who considered it as a distinct entity.[4] In 1969, Philipsen and Birn proposed the term AOT,[5] indicating that it was not a variant of ameloblastoma. The term AOT was accepted in the first WHO classification of odontogenic tumors established in 1971.[6] The term AOT seems to be the most appropriate, as these tumors unlike the ameloblastoma, are benign and present a very low recurrence, making it unnecessary to carry out extensive and aggressive surgery.[7] The surgical management of this lesion would be ennucleation along with the associated impacted tooth. There are three variants of AOT,[7–9] the follicular type (73%), which has a central lesion associated with an impacted tooth and is usually diagnosed as a dentigerous cyst or follicular cyst; the extra follicular type (24%) has a central lesion and no connection with the tooth, usually present as a unilocular well-defined radiolucency above or superimposed on the roots of erupted teeth and often resembling a residual globulomaxillary or lateral periodontal cyst. The peripheral type (3%) usually presents as a gingival swelling, located palatally or lingually relative to the involved tooth.

This is a report of a large follicular AOT associated with a dentigerous cyst in the posterior maxilla in association with an impacted second molar- a very rare occurrence, which was mistaken for a dentigerous cyst clinically and radiographically.

## CASE REPORT

**Figure 1:**
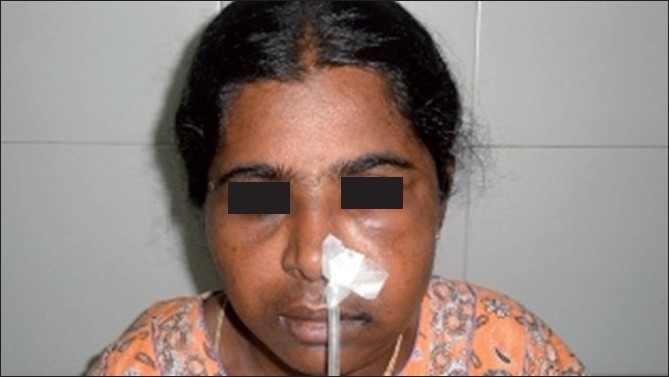
Patient postoperative

**Figure 2:**
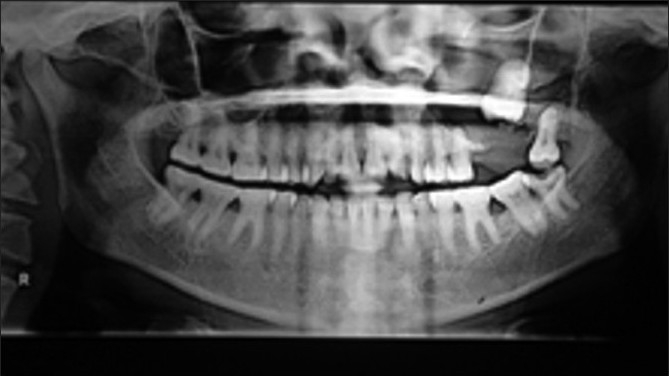
Orthopantomogram

A 39-year-old female patient reported to my dental clinic with a chief complaint of pain and swelling over left side of upper jaw of 1month duration with mobility of her upper last tooth. On examination of the patient, she had a diffuse extra-oral swelling measuring approximately 1.5×1.5cm extending from left infraorbital region superiorly to left corner of mouth, obliterating the nasolabial crease. There was no paraesthesia over infraorbital region. Intraoral examination revealed a soft fluctuant swelling on left side maxilla extending from left upper premolar to third molar region, obliterating the buccal vestibule. Left upper first and second molars were missing, left upper third molar showed grade 3 mobility. Mucosa overlying the swelling was normal. On aspiration, straw-colored fluid was obtained. Orthopantomogram was taken which revealed a well-defined, unilocular corticated radiolucency extending from left upper first premolar to third molar region, associated with an impacted second molar and involving the left maxillary antrum [Figures 1, 2]. On the basis of clinical and radiographical findings, the differential diagnosis were dentigerous cyst, unicystic ameloblastoma and AOT.

**Figure 3:**
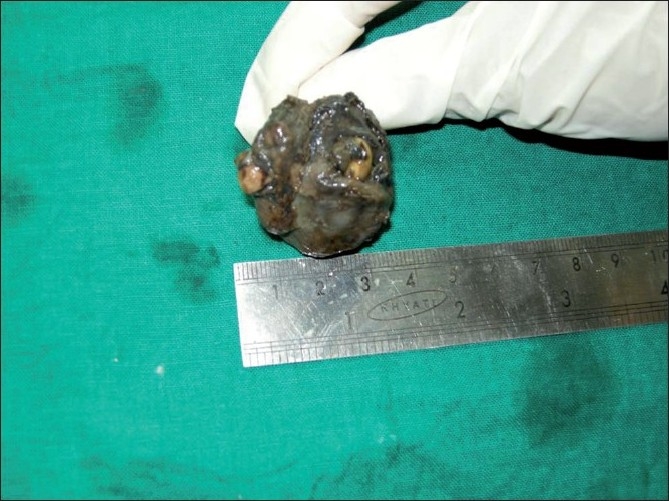
Postoperative specimen

**Figure 4:**
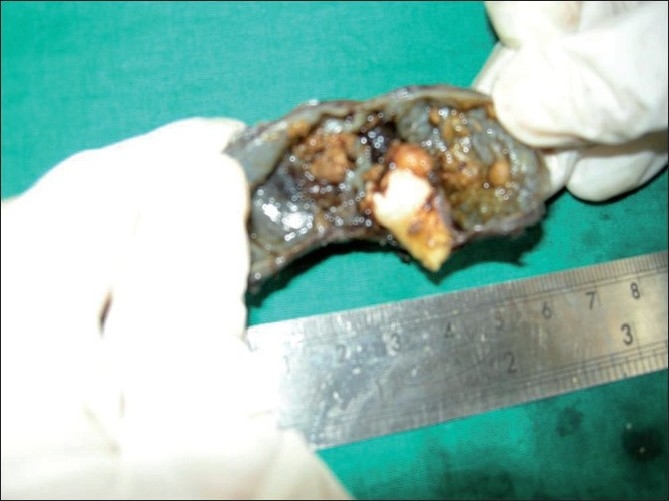
Impacted second molar tooth associated with lesion

The patient underwent surgery under general anesthesia. A mucoperiosteal flap was raised from the left canine to third molar region in the buccal vestibule. The buccal cortex was resorbed completely with thin shells of bone in between. The lining of the cystic lesion was carefully separated from the mucoperiosteam and the lesion was enucleated along with the impacted second molar and the mobile third molar [Figures 3 and 4]. The wound was irrigated with saline and betadine. Homeostasis was achieved. Nasal antrostomy was done and antral pack placed in situ. Wound was sutured with 3-ovicryl. Antral pack was removed after 48 hours [Figure 1]. Healing was uneventful and patient is disease-free for the last 6 months.

**Figure 5:**
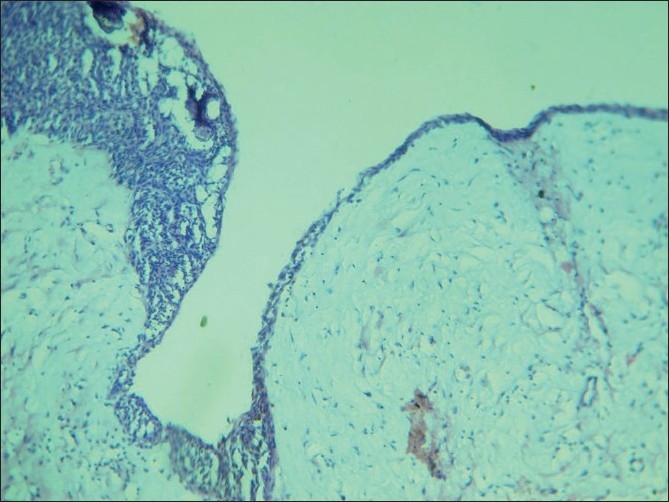
10× view showing a thin nonkeratinized lining epithelium continuous and showing proliferation in one area, spindle cells and calcifications are seen

**Figure 6:**
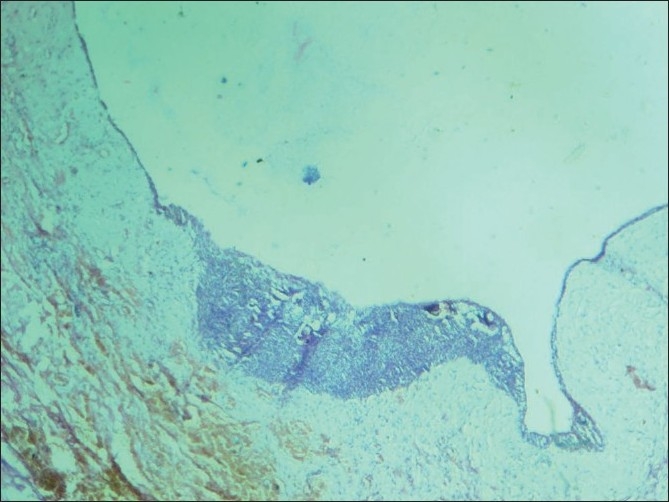
10× view showing a thin nonkeratinized lining epithelium proliferating into the lumen showing spindle cells and calcification

**Figure 7:**
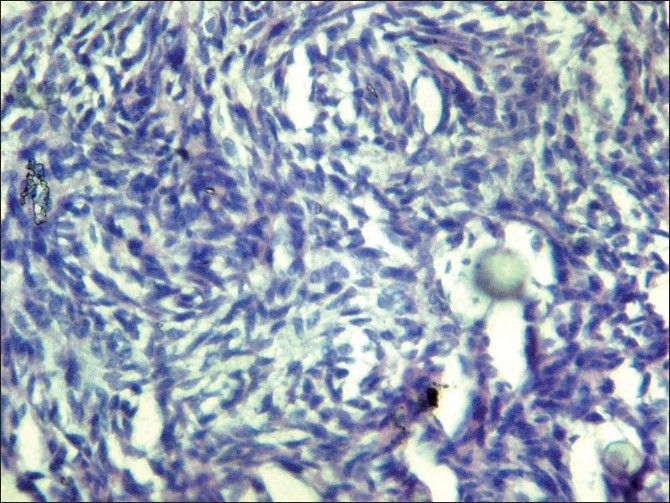
40× view showing spindle shaped cells in the form of whorls and rosette formation with calcification

Histopathological examination revealed thin nonkeratinized lining epithelium and shows proliferation into the lumen with spindle cells and calcifications [Figure 5]. The spindle-shaped cells are in the form of whorls and rosette formation with calcification [Figures 6 and 7]. The ductal like structures were surrounded by columnar epithelial cells and filled in some areas with eosinophilic material. In other places amorphous calcified material was present. The histopath ological report was that of AOT developing in a dentigerous cyst.

## DISCUSSION

Adenomatoid odontogenic tumor is a slow growing lesion, constituting only 3% of all odontogenic tumors[4] and 0.1% of tumors of jaws[10] in general with a predilection for the anterior maxilla (ratio 2:1 relative to mandible) usually associated with impacted canine, of young females in the second decade of life. In our case the lesion occurred in the posterior maxilla associated with an impacted second molar which is unusual. Till now, there is no reported case of AOT in relation with the second molar in the literature review. The patient in our report was in the fourth decade. The female to male ratio for all age groups and all variants is close to 2:1.[11] Usually, the tumors do not exceed 1-3 cm in greatest diameter. The lesions are usually asymptomatic, but may be associated with cortical expansion as in our case. The involved teeth are commonly impacted and adjacent teeth may be slightly displaced,[12] Root resorption is not a usual feature. The tooth that is impacted commonly is the canine in 40%.[13] Unerupted first and second molars are rarely involved, nor are deciduous teeth. But in our case, it is the second molar which is involved in the lesion. This uncommon tumor which occurs even less frequently than the odontoma, cementoma, myxoma and ameloblastoma has been considered a hamartoma rather than a neoplasm,[8] though there is an ongoing dispute regarding this.

Radiographically, this lesion usually surrounds an unerupted tooth and is seen as a corticate radiolucency with small radiopacities, but there are cases where the lesion has no radiopaque component, as in our case, and in such a case – a dentigerous cyst is the preferred differential diagnosis. However, an AOT often appears to envelop the crown as well as the root as shown in the picture of our case, where we divided the specimen to show the relation of the lesion to the tooth; unlike the dentigerous cyst which does not envelop the roots.[4,14–16] When we consider the pathogenesis of this tumor, the origin of AOT is controversial. Some believe they originate from the odontogenic epithelium of a dentigerous cyst. Santos *et al*.[17,18] reported a case of AOT being developed in the fibrous capsule of the dentigerous cyst. Garcia Pola *et al*.[18,19] described the proliferation of an AOT in the epithelial border of a dentigerous cyst. Cassiano Francisco Weege *et al*. reported a case of AOT associated with dentigerous cyst. The tumor in association with a dentigerous cyst is reported to occur in the anterior maxilla and other areas of the jaw such as the angle of the mandible, and in our case – the posterior maxilla.

Therefore, dental laminar remnants could probably be the progenitor cells for this benign odontogenic tumor. According to this hypothesis, the lesion grows next to or into a nearby dental follicle leading to the “envelopmental theory”.[2,20] In our case, the lesion surrounded the fully formed second molar tooth, which suggests an envelopmental pathogenesis. Recent reports indicate that the cells of an AOT usually differentiate toward an apparent ameloblastic phenotype but fail to achieve further functional maturation.[2,21] WHO has described the histological features of the tumor as “A tumor of odontogenic epithelium with duct-like structures and with varying degree of inductive changes in the connective tissue. The tumor may be partly cystic and in some cases the solid lesion may be present only as masses in the wall of a large cyst. It is generally believed that the lesion is not a neoplasm. Moreover, eosinophilic uncalcified amorphous material can be found and is called “tumor droplets”,[3] as is seen in the cut specimen of our case.

Immunohistochemical studies report that the slow growth, its benign character and low tendency to recur are clearly related to the low cellular proliferation observed on carrying out immunostaining for the Ki67 antigen. Only 2-3% of components of tumor demonstrated reactivity for Ki67 in a study done by Francisco Jose Vera Sempere *et al*.., a percentage similar to that recently indicated by Leon *et al*.[22] This positivity was found to be the sole center of the tumor proliferation. A second point of interest noted was the marked and diffuse positivity for AE 1-3 associated with a universal nuclear reactivity of the P63 antigen throughout practically all the cells forming this tumor. From the immunohistochemical point of view, this last aspect confirms the basal and/or progenitor (p63+) character of epithelial (AE1-3+) cells of the elements that make up this benign tumor of low-grade proliferation, although possessing a wide phenotypic morphological variety.[22]

Much of the above data (low cellular proliferative capacity, basal character – confirmed by the universal reactivity of p63 – of all the proliferating cells, the presence of spherical calcification as forms of abortive enamel, or the inadequate formation of dentinoid tissue, the existence of mesenchymal inductive changes with production of amyloid-like material) would seem to reinforce the idea of the hamartomatous character of thin infrequent form of benign, odontogenic tumor, as previously indicated by other authors, and indicating that this is probably not a true neoplastic growth.[5] Given its benign behavior, slow growth and clear delimitation, as well as its low tendency to recur, the treatment of choice is ennucleation and simple currettage, although in exceptional cases of large tumors or risk of bone fracture, partial resection, en bloc of the mandible or maxilla has been indicated.[23] Additionally, the use of lyophilized bone and guided tissue regeneration has been recommended in cases where surgical extirpation has left a large exposed osseous cavity.[23]

## CONCLUSION

This is a case report of AOT in association with a dentigerous cyst in the posterior maxilla associated with an impacted second molar, which is extremely rare. The article also provides a review of literature on the clinical, histopathological, radiographical details and immunohistochemical studies of the lesion.
